# Assembly of Designer TAL Effectors by Golden Gate Cloning

**DOI:** 10.1371/journal.pone.0019722

**Published:** 2011-05-19

**Authors:** Ernst Weber, Ramona Gruetzner, Stefan Werner, Carola Engler, Sylvestre Marillonnet

**Affiliations:** Icon Genetics GmbH, Halle/Saale, Germany; Ecole Normale Superieure, France

## Abstract

Generation of customized DNA binding domains targeting unique sequences in complex genomes is crucial for many biotechnological applications. The recently described DNA binding domain of the transcription activator-like effectors (TALEs) from *Xanthomonas* consists of a series of repeats arranged in tandem, each repeat binding a nucleotide of the target sequence. We present here a strategy for engineering of TALE proteins with novel DNA binding specificities based on the 17.5 repeat-containing AvrBs3 TALE as a scaffold. For each of the 17 full repeats, four module types were generated, each with a distinct base preference. Using this set of 68 repeat modules, recognition domains for any 17 nucleotide DNA target sequence of choice can be constructed by assembling selected modules in a defined linear order. Assembly is performed in two successive one-pot cloning steps using the Golden Gate cloning method that allows seamless fusion of multiple DNA fragments. Applying this strategy, we assembled designer TALEs with new target specificities and tested their function *in vivo*.

## Introduction

The development of synthetic nucleases that cleave unique genomic sequences in living cells provides powerful tools for genome engineering, allowing targeted gene knockout and gene replacement [1]. A key component of these artificial nucleases is the DNA binding domain which directs the nuclease to its target sequence. To date, the majority of customized DNA targeting domains used for genome engineering that have been made are based on engineered zinc-finger domains. However, the creation of new DNA binding specificities has proven to be technically challenging and time consuming. An alternative to zinc-finger domains may be the recently described DNA binding domain found in transcription activator-like effectors (TALEs) [2], [3]. TALEs are virulence factors of plant pathogens from the genus *Xanthomonas* that are translocated via a type III secretion system inside the plant cell. The TALEs are then imported into the nucleus, where they bind to specific DNA sequences and transcriptionally activate gene expression [4], [5]. DNA binding is mediated by a central repetitive region, formed by up to 33 tandem repeats of a 33 to 35 amino acid motif, each repeat corresponding to one DNA base pair of the target sequence. The amino acid sequences of the repeats are nearly identical, beside amino acid positions 12 and 13, the so-called repeat variable diresidues (RVD). Repeats with different RVDs show different DNA base pair preferences, and consecutive RVDs in a TALE correspond directly to the DNA sequence in the binding side, resulting in a simple one-repeat-to-one-base pair code [6], [7]. Knowledge of this TALE recognition code has been used to predict the DNA binding specificity of native TALEs and to create designer TALEs (dTALEs) which transcriptionally activate user-defined promoter sequences [8], [9]. Furthermore, several groups have combined dTALE DNA binding domains with the FokI derived DNA-cleavage domain, resulting in potent tools for genome engineering [10], [11], [12], [13]. However, assembly of multiple repeats with highly identical sequences by standard cloning approaches is challenging and chemical synthesis of the entire repeat region expensive.

We present here an approach to assemble genes encoding TALE repeat domains based on the scaffold of AvrBs3, the first described and well characterized TALE family member [14]. For each of the 17 full repeats found in AvrBs3, four module types were generated, each with preference to one of the four DNA base pairs. With this set of 68 repeat modules, DNA recognition domains for any 17 nucleotide target sequence of choice can be assembled in two cloning steps. Both cloning steps use the Golden Gate cloning method that allows directional and seamless assembly of multiple DNA fragments [15], [16]. As a proof of principle, we created three dTALE proteins designed to target the promoter of a reporter construct stably integrated in the *Nicotiana benthamiana* genome, and show that all three dTALEs are able to activate the reporter construct.

## Results

### dTALE assembly strategy

**Figure 1:**
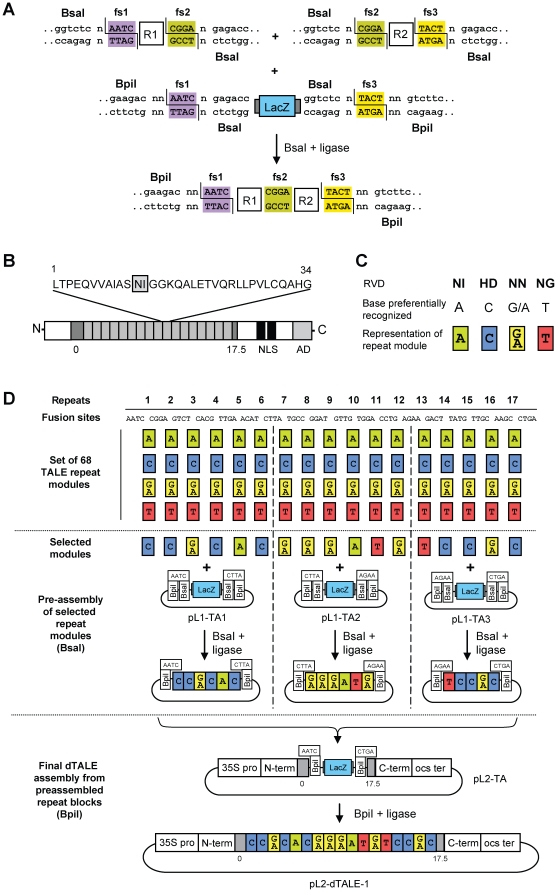
General overview of the two-step cloning strategy for dTALEs assembly. (A) Golden Gate cloning principle applied for assembly of dTALEs. Plasmids encoding selected repeat modules (an example with only two modules, R1 and R2, is shown here due to space limitation) are mixed in one tube together with BsaI, T4 DNA ligase and the destination vector (containing a *lacZα* fragment for blue-white selection). Assembly of R1 and R2 using BsaI and ligase gives rise to a plasmid lacking the initial BsaI sites, but containing a block of assembled repeats flanked by two BpiI sites. The two BpiI sites allow release of the assembled repeats as one block for the second step of cloning. fs, fusion site. (B) Structure of AvrBs3. AvrBs3 contains a central region with 17 direct repeats (light grey boxes) flanked by a thymidine-specific repeat (repeat 0) and a half repeat (repeat 17.5, both flanking repeats shown as dark grey boxes). Two nuclear localization sequences (NLS, black bars) and a transcription activation domain (AD) are located in the C-terminal region. One representative 34 aa repeat is shown, with the RVD of the NI type highlighted in grey. (C) RVD types and their specificities. (D) Set of 68 repeat modules, with 4 modules with different specificities for each of the 17 repeat positions. Repeat modules are flanked by two BsaI sites with fusion sites selected from the codon-optimized sequence of AvrBs3 (see **Supporting Information S1**). Sets of five (for repeats 13–17) or six (for repeats 1–6 and 7–12) selected repeat modules are preassembled via BsaI into preassembly vectors (pL1-TA1 to 3). Preassembled repeat blocks are then combined in the final destination vector (pL2-TA) using a second BpiI-based Golden Gate cloning reaction. Construction of dTALE-1 is shown as an example.

The dTALE assembly strategy described here uses the Golden Gate cloning method, which is based on the ability of type IIS enzymes to cleave outside of their recognition site. When type IIS recognition sites are placed to the far 5' and 3' end of any DNA fragment in inverse orientation, they are removed in the cleavage process, allowing two DNA fragments flanked by compatible sequence overhangs, termed fusion sites, to be ligated seamlessly (**Fig. 1A**). Since type IIS fusion sites can be designed to have different sequences, directional assembly of multiple DNA fragments is feasible [17]. Since the type IIS restriction sites used for assembly are removed in the cloning process, restriction and ligation can be performed together, allowing continuous redigestion of unwanted ligation products and increasing formation of the only stable ligation product, which is the desired construct [15]. Using this strategy, up to 9 DNA fragments can be assembled from undigested input plasmids in a one-pot reaction with high efficiency [16].

We chose the native TALE AvrBs3 as a scaffold for customized assembly of dTALE constructs. The central DNA binding domain of AvrBs3 is formed by 17.5 tandemly arranged 34 amino acid repeats, with the last half repeat showing similarity to only the first 20 amino acids of a full repeat. In addition to the 17.5 repeats, AvrBs3 contains an N-terminally adjacent repeat 0 that is thought to be specific for a thymidine (as *Xanthomonas* TALE binding sites always have thymidines at the position corresponding to repeat 0 [6], [7]) (**Fig. 1B**). To reduce the risk of recombination events between the 17.5 highly homologous repeat sequences, we codon-optimized *avrBs3* applying the *Nicotiana tabacum* codon usage. From this modified DNA sequence, we selected 18 fusion sites that artificially define the ends of the 17 repeat modules that will be used for assembly (**Fig. 1D** and **Supporting Information S1**). For each of the 17 repeat modules, we designed four variants with different RVDs, each with a different DNA base pair preference. We used the most abundant RVDs found in native TAL effectors (NI for A, HD for C, NN for G and NG for T) (**Fig. 1C**) [6], [7]. However, only the RVD types NI, HD, and NG show a high specificity for their target nucleotide, whereas the RVD NN targets G and A. The designed repeat modules were then constructed from two overlapping oligonucleotides (see methods section). Each resulting module is flanked by two fusion sites and two external BsaI recognition site sequences, as illustrated in **figure 1A**. The complete set contains 68 sequenced TALE repeat modules (**Fig. 1D**).

Although 9 DNA fragments can be efficiently assembled in a single Golden Gate cloning reaction, cloning efficiency is significantly reduced for assembly of 17 repeat modules in a single cloning reaction (0 to 3 colonies out of 12). Therefore, we split the assembly in two successive steps. In the first cloning step, blocks of 5 or 6 repeats are assembled in three preassembly vectors, one for repeat module positions 1–6, one for positions 7–12 and one for positions 13–17 (pL1-TA1 to 3). The preassembly vectors confer ampicillin resistance (Ap^R^) and encode a *lacZ*α fragment for blue/white selection. On both sides of the *lacZ*α fragment two different type IIS recognition sequences - BsaI and BpiI - are positioned in inverse orientation relative to each other, but creating the identical fusion site (**Fig. 1A**). After preassembly of the 3 repeat blocks using BsaI, the intermediate blocks are released via BpiI and cloned into the final assembly vector (pL2-TA). pL2-TA confers kanamycin (Km^R^) resistance to counterselect against the plasmid backbones of the preassembly vectors (Ap^R^), and allows plasmid replication in *E.coli* and *Agrobacterium*. The vector pL2-TA also contains all elements of the final dTALE expression construct, except the repeat modules (**Fig. 1D**). In particular, it contains a promoter and terminator required for expression in plant cells, as well as the N- and C-terminal domains of AvrBs3, including the unmodified repeats 0 and 17.5 flanking the two BpiI sites used for insertion of the lacking repeat blocks.

### Assembly of dTALEs and their functional testing

**Figure 2:**
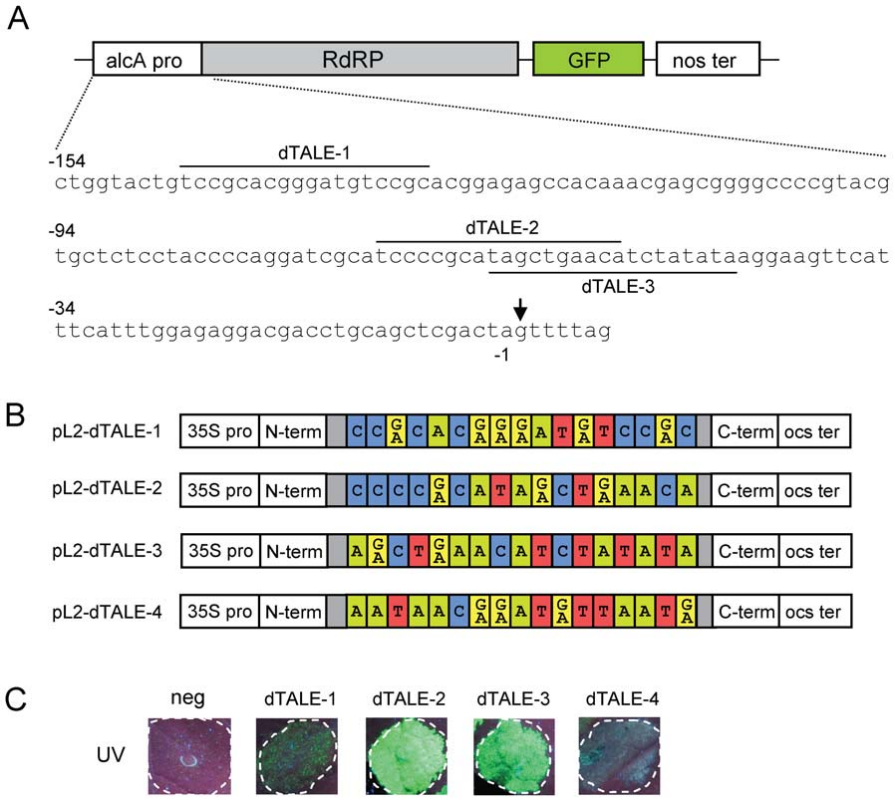
Design and functional test of customized TAL effectors. (A) Structure of the reporter construct present in transgenic *N. benthamiana* plants. The reporter construct contains a TMV-based viral vector construct under control of the alcA promoter. The vector contains the RNA-dependent RNA polymerase (RdRp) and a GFP gene, but lacks the viral movement and coat protein genes. Viral vector-mediated GFP expression is obtained only in cells where the alcA promoter has been activated. Sequences selected for engineering of dTALE-1 to dTALE-3 are indicated by a black line. The transcription start site of the TMV-based vector is marked by an arrow. (B) Schematic representation of dTALE-1 to 4 constructs. (C) *Agrobacterium tumefaciens* strains containing dTALE-1 to dTALE-4 constructs were inoculated into leaves of transgenic plants. An empty *Agrobacterium* strain was also inoculated as a negative control (neg). GFP expression was analyzed 5 days after inoculation under UV light. dTALE-1, 2 and 3, which target sequences in the alcA promoter, induced GFP expression. In contrast, dTALE-4, which targets a randomly selected sequence (not present in the promoter), did not induce any GFP expression.

To test functionality of the assembled dTALEs, we used transgenic *N. benthamiana* plants containing a stably integrated GFP reporter construct (**Fig. 2A**). This construct consists of a tobacco mosaic virus-based viral vector under control of the alcA promoter from *Aspergillus nidulans* [18], [19]. Since the alcR transcriptional activator that is required for activation of the alcA promoter is not present in the transgenic plants, the alcA promoter can be considered here as a minimal promoter. Three sequences were chosen from the promoter, all starting with a thymidine as defined by the specificity of repeat 0. The target sequence chosen for dTALE-1 is overlapping with the alcR binding site in the alcA promoter (bp -143 to -127), while the target sequences for dTALE-2 and dTALE-3 consist of bp -61 to -45 and bp -69 to -53 respectively (target site positions numbered relative to the viral vector transcription start, **Fig. 2A**). dTALE-4 was constructed as a negative control and targets a randomly selected sequence not found in the promoter region.

For construction of the 4 dTALE constructs, 12 parallel BsaI-based Golden Gate cloning reactions were set up with selected modules and the respective preassembly vectors pL1-TA1 to 3. For each reaction, plasmid DNA from two colonies was purified and sequenced, and all plasmids were found to contain the correct sequence. Preassembled repeat blocks were assembled to the final constructs dTALE-1 to 4 using a second BpiI-based Golden Gate cloning reaction (**Fig. 2B**). Eleven out of 12 colonies analyzed contain a correct construct. After sequence verification, the constructs dTALE-1 to 4 were transformed into *A. tumefaciens* and inoculated into leaves of transgenic *N. benthamiana* plants containing the GFP reporter construct. All three dTALEs with DNA binding domains designed to target sequences in the alcA promoter induced GFP expression in infiltrated leaf areas, with expression from dTALE-1 being the weakest. In contrast, dTALE-4 did not induce any GFP expression from the reporter construct (**Fig. 2C**).

## Discussion

We have shown here that constructs for dTALE proteins containing a 19 base DNA binding domain (consisting of 17 engineered full repeats, repeat 0 and the half repeat 17.5) can be easily assembled by two successive one-pot Golden Gate cloning reactions. We have prepared a set of 68 repeat modules that allows construction of DNA binding domains for any 17 base user-defined target sequence. The native half repeat 17.5 of AvrBs3, which contains a RVD specific for thymidine, was included in the C-terminal fragment of the final assembly vector. It would however be possible to also make half repeat modules with different RVD types to improve the binding of dTALE proteins for target sequences that do not have a T at this position. Such repeats could be assembled together with repeats 13 to 17 in a new preassembly vector replacing pL1-TA3. A new compatible final assembly vector lacking the half repeat should also be made.

In case 17 repeats are not sufficient to provide specific binding, dTALE proteins with additional repeats could easily be constructed. In order to expand the TALE modular cloning system to more than 17 repeats, new unique fusion sites have to be defined for each additional repeat, and one or more new preassembly vectors specific for the added fusion sites have to be constructed. A further option to increase dTALE specificity is the replacement of the NN RVD, which has an equal preference to A and G, by the highly G-specific NK RVD [9], [12].

The Golden Gate cloning method provides a perfect fit for dTALE protein engineering because it allows directional and seamless assembly of multiple DNA fragments. In addition, this cloning method is sequence-independent and allows assembly of repeats with identical or highly homologous sequences, since only the 4 base pair fusion sites at the end of the repeats have to be unique. Selection of fusion sites with unique sequence at the ends of successive repeats can be easily accomplished by either changing the codon usage of the ends of the repeats, or by shifting the fusion sites a few nucleotides at the ends of the various repeats. Since a complementary shift can be selected at the beginning of each following repeat (as shown in the result section/supporting information), seamless assembly of direct repeats can then be easily achieved.

Other alternative methods for seamless assembly of multiple DNA fragments include SLIC [20], SOEing [21] and ssDNA oligonucleotide assembly [22]. These methods are however limited by the homology present among the repeats since they either involve PCR steps [21] or require annealing of single-stranded DNA fragments [20], [22], both of which run the risk of deleting some of the repeats by recombination during amplification and/or cloning. Codon optimization may nevertheless be used to minimize the risk of loss of repeats during cloning. A recently published protocol combines the use of type IIS enzymes and PCR amplification of codon-optimized repeats, and was shown to allow assembly of dTALEs containing 12.5 repeats [23]. This protocol is however more laborious, as it requires two rounds of PCR amplification and several purification steps and, since PCR is involved, some of the final constructs may be expected to contain mutations derived either from polymerase amplification or from the primers.

In conclusion, the cloning system described here provides a simple and economical way of assembling constructs encoding dTALE proteins for genome engineering and other biotechnological applications.

## Methods

### Molecular biology reagents

Restriction enzymes used in this study were purchased from New England Biolabs (Ipswitch, MA) and Fermentas (Burlington, Canada). T4 DNA ligase was purchased from Promega (Fitchburg, WI). Plasmid DNA preparations were made by using the NucleoSpin Plasmid Quick Pure kit (Macherey-Nagel, Düren, Germany) following the manufacturer protocol. Plasmid DNA concentration was measured using a Nano Drop® Spectrophotometer ND-2000 (Peqlab, Erlangen, Germany). DNA sequences for the AvrBs3 N- and C-termini were codon-optimized using the *Nicotiana tabacum* codon usage (GENEius software from MWG Eurofins, Ebersberg, Germany) and were synthesized by this company. Both synthesized fragments do not contain any BpiI or BsaI restriction sites. Sequences of the codon-optimized *avrBs3* gene and of the 68 repeat modules, as well as primer sequences necessary for construction of the destination plasmids are listed in **Supporting Information S1**.

### Vector construction

The repeat modules were made by annealing two partially overlapping primers and filling the single-stranded extensions using KOD polymerase (Merck, Darmstadt, Germany). The double-stranded products were digested with XhoI and cloned in the SalI site of a pUC19-derived vector conferring spectinomycin resistance and lacking BpiI and BsaI sites. For construction of the preassembly vectors pL1-TA1 to 3, a *lacZ*α fragment was amplified using primers ecvprac1/11, ecvprac18/19 and ecvprac23/24 (sequences given in **Supporting Information S1**). The PCR products were cloned via DraIII in a pUC19-derived plasmid conferring ampicillin resistance. The final destination plasmid pL2-TA was assembled with the modular cloning system described in [24]. The 35S promoter module, the synthesized AvrBs3 N- and C-termini, a *lacZ*α module and an ocs terminator were assembled via a BsaI-based Golden Gate cloning reaction in pL1F-1. The complete cassette was then transferred to the vector pL2-1 conferring kanamycin resistance using BpiI.

### Standard Golden Gate assembly reaction protocol

One-step one-pot restriction/ligations were set up using approximately 30 fmol (∼100 ng for a 5 kb plasmid) of each plasmid in a mix containing Promega ligation buffer, 10 U of the selected restriction enzyme (BsaI or BpiI) and 10 U T4 DNA ligase, in a final reaction volume of 20 µl. The reactions were incubated for 2 hours at 37°C, 5 minutes at 50°C and 5 minutes at 80°C. The mix was then added to 100 µl chemical competent DH10b cells, incubated for 30 min on ice and transformed by heat shock. Two clones were analyzed by restriction analysis and, optionally, sequencing.

## Supporting Information

Supporting Information S1 Sequence of the codon-optimized *avrBs3* gene and of the primers required for synthesis of the TALE repeats and of the preassembly vectors. (A) Sequence of the codon-optimized *avrBs3* gene. The sequences selected as fusion sites for assembly of dTALEs are shown in bold and underlined. (B) Primer sequences required for TALE repeat construction. (C) Primer sequences for construction of preassembly vectors pL1-TA1-3.
(DOC)
