# Supplementary material for: Assembly of Designer TAL Effectors by Golden Gate Cloning
Source: PLoS One. 2011 May 19;6(5):e19722. doi: 10.1371/journal.pone.0019722 (PMC3098256; doi:10.1371/journal.pone.0019722)
Supplement: Supporting Information S1 — Sequence of the codon-optimized avrBs3 gene and of the primers required for synthesis of the TALE repeats and of the preassembly vectors. (A) Sequence of the codon-optimized avrBs3 gene. The sequences selected as fusion sites for assembly of dTALEs are shown in bold and underlined. (B) Primer sequences required for TALE repeat construction. (C) Primer sequences for construction of preassembly vectors pL1-TA1-3. (DOC) [file pone.0019722.s001.doc]

**Supplementary Information SI**

**A. Sequence of the codon-optimized avrBs3 gene with highlighted fusion site sequences.**

atggaccccattcggtcaaggacgccatctccggctcgggaacttctacctggaccacaacccgatggggttcagcctacggccgataggggggtatcaccccccgcgggcggccccttggatggacttcccgcacgtcgaacgatgagtcgtacacgactaccgagcccgcctgcaccttctccagcattttctgctggctcttttagcgacctcctacgccagttcgacccatcattattcaatacatcccttttcgattctctcccccctttcggtgcacatcatacagaggctgcaactggcgaatgggatgaggtccaatctggtctgagggccgctgacgcaccccctccaaccatgagagttgctgtgactgctgcacgtcctccccgtgcgaaaccagcacctagacgacgtgcggcacagccttcggacgctagccctgctgcccaggttgatctcaggacactcggctacagtcaacagcaacaggagaaaataaaaccgaaagtccgatcaacggttgctcaacaccatgaggctctcgtgggtcatggctttacacatgctcacatcgttgcgctatcgcaacatcccgctgcacttggaaccgtcgcagttaaatatcaagatatgattgcagcactacctgaggctacacatgaagcaatcgtgggcgttggtaagcagtggtccggagctcgggcgttggaagcccttctcactgtagccggcgagttacgcggtccccctcttcagttggacactggtcaattgctcaagatcgcgaagcgtggaggagtgaccgcggtagaggcagtacacgcttggagaaatgccctgacaggcgctccatta**AAT**

repeat 1

**C**tcacgccagaacaagtggtcgctatcgcctcccatgacggtggaaaacaagcactagaaactgtccaaagattattgcctgttctttgtcaggcaca**CGGA**

repeat 2

cttaccccacaacaagtcgttgctatagcctccaacggtggagggaagcaagcgttagaaacagtgcagcggctactccctgtattatgccaggctcatg**GT**

repeat 3

**CT**aactccacaacaagtggtggctatagcctcaaattcgggtggtaaacaggcacttgaaaccgtccaaagactcctgccggtcctctgccaggca**CACG**gc

repeat 4

ctcacccccgaacaagtggtggctattgcttcgaatggcggaggtaagcaggctttagagacagtccagagactactacccgttctatgccaggcccatggt

repeat 5

**TTGA**ccccggaacaggttgttgctattgcgtcaaatattggcggcaagcaagcgttggaaaccgttcaagcattactccctgttctctgtcaagc**ACAT**ggg

repeat 6

ctaacgcccgagcaggttgttgcaattgcatcaaatattggaggaaagcaggccttagaaacggtacaggcacttttaccagtcctttgccaagcacacggg

repeat 7

**CTTA**cacccgaacaagtggtcgctattgcaagtaatatcggtggaaaacaagcactggaaaccgtgcaggcgcttttgccggtatta**TGCC**aagctcacggc

repeat 8

ctaactcctgaacaggtggttgcgattgcctcacatgatgggggtaaacaggcactggagactgtgcagcggcttttgcctgttttgtgtcaagctcat**GGA**

repeat 9

**T**tgacaccagagcaggtggtcgctatagctagtcatgatggaggtaaacaagcgcttgaaaccgtgcaacgtctgctgccagttctatgtcaagctcatgg**G**

repeat 10

**TTG**accccacaacaggttgtagcgatcgcttccaacggcggaggaaagcaagctctagaaacggtgcagaggctcctcccggttctttgtcaggcgca**TGGA**

repeat 11

ttgaccccggagcaggtggtcgcaatcgccagtaactcgggaggtaagcaggccttggaaaccgttcaggcgttactcccggttctatgccaggcgcatgg**C**

repeat 12

**CTG**acccctgaacaggttgtggcgatagccagtaactccgggggaaagcaggcacttgaaaccgtacaacgactcctcccagtcctttgtcaagcccacgga

repeat 13

ttgactcc**AGAA**caagtagttgctatagcttcgcatgatggaggaaagcaggcccttgaaacagttcagcgtcttttgccagtgttgtgtcaagcacacgga

repeat 14

tt**GACT**cctgaacaggttgtcgccattgcatctcacgatggtggtaagcaagctctcgaaaccgtacagcgactcttgcctgttc**TATG**ccaagcgcatggc

repeat 15

ttgacgccggaacaggtggtagccatagcaagccacgatggtggcaaacaagctcttgaaacagttcaaaggttgttacctgtgct**TTGC**caagcccacggt

repeat 16

ttgacccctcaacaggtggttgctatagcatcaaatggcgggggacggcctgctcttgagacagtgcagcgcctgttgcccgtgttgtgtc**AAGC**gcatggc

repeat 17

ttaacaccggaacaggtcgtggcaattgcgtcacacgatggcggcaaacaagcgctggaaaccgttcagcgactcttgcctgttctgtgccaagctcacggt

**CTGA**cgccccaacaggttgttgccattgcttcaaatggaggagggaggccagcccttgagtcgattgtcgcacagctatctcggcccgaccctgctttagccgctctgacaaatgatcatcttgtggctctcgcctgcttaggaggtcgcccagctttagacgcagtaaaaaagggtctacctcatgctccggccttaatcaagaggacgaatcgtagaatcccagaacgaacgagccatcgcgtagccgatcacgctcaagttgttagggttttaggtttttttcagtgtcattcacatccggcacaagctttcgatgatgccatgacccagtttggtatgtcaaggcatggattactgcaacttttcagaagagtaggagtgacagagctcgaagccagaagcggaactctgccacccgctagccaaagatgggataggatattgcaggcgagtggaatgaagcgcgcgaaaccatctccaacaagcactcaaaccccggatcaagcgagtttgcacgctttcgcagattctctcgaacgagatttggatgccccttctccaatgcacgaaggtgatcaaactagggcgagtagcaggaagaggtctaggagtgatcgtgcagttacgggcccctcagcacaacagtcttttgaggtcagggtgccagaacaaagggacgctttacatctcccattgtcttggcgtgtaaaaaggccgcgaactagtattggagggggattaccggacccagggacccccactgctgctgatctagctgcttctagtacggtaatgcgcgagcaagacgaggatccatttgctggggcagctgatgacttccccgcattcaacgaagaagaattagcatggttgatggagttactgccacagtaa

**B. Primer sequences for TALE repeat construction**

A3R1R

ttctcgaggtctcatccgtgtgcctgacaaagaacaggcaataatctttggacagtttctagtgcttgttttccacc

A3R1A

ttctcgaggtctcaaatctcacgccagaacaagtggtcgctatcgcctccaatattggtggaaaacaagcactagaa

A3R1C

ttctcgaggtctcaaatctcacgccagaacaagtggtcgctatcgcctcccatgatggtggaaaacaagcactagaa

A3R1G

ttctcgaggtctcaaatctcacgccagaacaagtggtcgctatcgcctccaataatggtggaaaacaagcactagaa

A3R1T

ttctcgaggtctcaaatctcacgccagaacaagtggtcgctatcgcctccaatggtggtggaaaacaagcactagaa

A3R2R

ttctcgaggtctcaagaccatgagcctggcataatacagggagtagccgctgcactgtttctaacgcttgcttccctcc

A3R2A

ttctcgaggtctcacggacttaccccacaacaagtcgttgctatagcctccaatatcggagggaagcaagcgttagaa

A3R2C

ttctcgaggtctcacggacttaccccacaacaagtcgttgctatagcctcccatgatggagggaagcaagcgttagaa

A3R2G

ttctcgaggtctcacggacttaccccacaacaagtcgttgctatagcctccaataacggagggaagcaagcgttagaa

A3R2T

ttctcgaggtctcacggacttaccccacaacaagtcgttgctatagcctccaatggtggagggaagcaagcgttagaa

A3R3R

ttctcgaggtctcacgtgtgcctggcagaggaccggcaggagtctttggacggtttcaagtgcctgtttaccacc

A3R3A

ttctcgaggtctcagtctaactccacaacaagtggtggctatagcctcaaacataggtggtaaacaggcacttgaa

A3R3C

ttctcgaggtctcagtctaactccacaacaagtggtggctatagcctcacacgatggtggtaaacaggcacttgaa

A3R3G

ttctcgaggtctcagtctaactccacaacaagtggtggctatagcctcaaataatggtggtaaacaggcacttgaa

A3R3T

ttctcgaggtctcagtctaactccacaacaagtggtggctatagcctcaaacggaggtggtaaacaggcacttgaa

A3R4R

ttctcgaggtctcatcaaaccatgggcctggcatagaacgggtagtagtctctggactgtctctaaagcctgcttacctcc

A3R4A

ttctcgaggtctcacacggcctcacccccgaacaagtggtggctattgcttcgaatattggaggtaagcaggctttagag

A3R4C

ttctcgaggtctcacacggcctcacccccgaacaagtggtggctattgcttcgcatgatggaggtaagcaggctttagag

A3R4G

ttctcgaggtctcacacggcctcacccccgaacaagtggtggctattgcttcgaataacggaggtaagcaggctttagag

A3R4T

ttctcgaggtctcacacggcctcacccccgaacaagtggtggctattgcttcgaatggtggaggtaagcaggctttagag

A3R5R

ttctcgaggtctcaatgtgcttgacagagaacagggagtaatgcttgaacggtttccaacgcttgcttgccgcc

A3R5A

ttctcgaggtctcattgaccccggaacaggttgttgctattgcgtcaaatattggcggcaagcaagcgttggaa

A3R5C

ttctcgaggtctcattgaccccggaacaggttgttgctattgcgtcacacgatggcggcaagcaagcgttggaa

A3R5G

ttctcgaggtctcattgaccccggaacaggttgttgctattgcgtcaaataatggcggcaagcaagcgttggaa

A3R5T

ttctcgaggtctcattgaccccggaacaggttgttgctattgcgtcaaatggaggcggcaagcaagcgttggaa

A3R6R

ttctcgaggtctcataagcccgtgtgcttggcaaaggactggtaaaagtgcctgtaccgtttctaaggcctgctttcctcc

A3R6A

ttctcgaggtctcaacatgggctaacgcccgagcaggttgttgcaattgcatcaaacatcggaggaaagcaggccttagaa

A3R6C

ttctcgaggtctcaacatgggctaacgcccgagcaggttgttgcaattgcatcacatgatggaggaaagcaggccttagaa

A3R6G

ttctcgaggtctcaacatgggctaacgcccgagcaggttgttgcaattgcatcaaataacggaggaaagcaggccttagaa

A3R6T

ttctcgaggtctcaacatgggctaacgcccgagcaggttgttgcaattgcatcaaacggaggaggaaagcaggccttagaa

A3R7R

ttctcgaggtctcaggcataataccggcaaaagcgcctgcacggtttccagtgcttgttttccacc

A3R7A

ttctcgaggtctcacttacacccgaacaagtggtcgctattgcaagtaatataggtggaaaacaagcactggaa

A3R7C

ttctcgaggtctcacttacacccgaacaagtggtcgctattgcaagtcatgatggtggaaaacaagcactggaa

A3R7G

ttctcgaggtctcacttacacccgaacaagtggtcgctattgcaagtaataatggtggaaaacaagcactggaa

A3R7T

ttctcgaggtctcacttacacccgaacaagtggtcgctattgcaagtaatggtggtggaaaacaagcactggaa

A3R8R

ttctcgaggtctcaatccatgagcttgacacaaaacaggcaaaagccgctgcacagtctccagtgcctgtttaccccc

A3R8A

ttctcgaggtctcatgccaagctcacggcctaactcctgaacaggtggttgcgattgcctcaaatattgggggtaaacaggcactggag

A3R8C

ttctcgaggtctcatgccaagctcacggcctaactcctgaacaggtggttgcgattgcctcacatgatgggggtaaacaggcactggag

A3R8G

ttctcgaggtctcatgccaagctcacggcctaactcctgaacaggtggttgcgattgcctcaaataacgggggtaaacaggcactggag

A3R8T

ttctcgaggtctcatgccaagctcacggcctaactcctgaacaggtggttgcgattgcctcaaatggtgggggtaaacaggcactggag

A3R9R

ttctcgaggtctcacaacccatgagcttgacatagaactggcagcagacgttgcacggtttcaagcgcttgtttacctcc

A3R9A

ttctcgaggtctcaggattgacaccagagcaggtggtcgctatagctagtaacattggaggtaaacaagcgcttgaa

A3R9C

ttctcgaggtctcaggattgacaccagagcaggtggtcgctatagctagtcatgatggaggtaaacaagcgcttgaa

A3R9G

ttctcgaggtctcaggattgacaccagagcaggtggtcgctatagctagtaataatggaggtaaacaagcgcttgaa

A3R9T

ttctcgaggtctcaggattgacaccagagcaggtggtcgctatagctagtaacggaggaggtaaacaagcgcttgaa

A3R10R

ttctcgaggtctcatccatgcgcctgacaaagaaccgggaggagcctctgcaccgtttctagagcttgctttcctcc

A3R10A

ttctcgaggtctcagttgaccccacaacaggttgtagcgatcgcttccaatatcggaggaaagcaagctctagaa

A3R10C

ttctcgaggtctcagttgaccccacaacaggttgtagcgatcgcttcccacgatggaggaaagcaagctctagaa

A3R10G

ttctcgaggtctcagttgaccccacaacaggttgtagcgatcgcttccaataacggaggaaagcaagctctagaa

A3R10T

ttctcgaggtctcagttgaccccacaacaggttgtagcgatcgcttccaatggcggaggaaagcaagctctagaa

A3R11R

ttctcgaggtctcacaggccatgcgcctggcatagaaccgggagtaacgcctgaacggtttccaaggcctgcttacctcc

A3R11A

ttctcgaggtctcatggattgaccccggagcaggtggtcgcaatcgccagtaatataggaggtaagcaggccttggaa

A3R11C

ttctcgaggtctcatggattgaccccggagcaggtggtcgcaatcgccagtcatgatggaggtaagcaggccttggaa

A3R11G

ttctcgaggtctcatggattgaccccggagcaggtggtcgcaatcgccagtaataatggaggtaagcaggccttggaa

A3R11T

ttctcgaggtctcatggattgaccccggagcaggtggtcgcaatcgccagtaatggtggaggtaagcaggccttggaa

A3R12R

ttctcgaggtctcattctggagtcaatccgtgggcttgacaaaggactgggaggagtcgttgtacggtttcaagtgcctgctttccccc

A3R12A

ttctcgaggtctcacctgacccctgaacaggttgtggcgatagccagtaacattgggggaaagcaggcacttgaa

A3R12C

ttctcgaggtctcacctgacccctgaacaggttgtggcgatagccagtcacgatgggggaaagcaggcacttgaa

A3R12G

ttctcgaggtctcacctgacccctgaacaggttgtggcgatagccagtaacaatgggggaaagcaggcacttgaa

A3R12T

ttctcgaggtctcacctgacccctgaacaggttgtggcgatagccagtaacggcgggggaaagcaggcacttgaa

A3R13R

ttctcgaggtctcaagtcaatccgtgtgcttgacacaacactggcaaaagacgctgaactgtttcaagggcctgctttcctcc

A3R13A

ttctcgaggtctcaagaacaagtagttgctatagcttcgaatattggaggaaagcaggcccttgaa

A3R13C

ttctcgaggtctcaagaacaagtagttgctatagcttcgcatgatggaggaaagcaggcccttgaa

A3R13G

ttctcgaggtctcaagaacaagtagttgctatagcttcgaataatggaggaaagcaggcccttgaa

A3R13T

ttctcgaggtctcaagaacaagtagttgctatagcttcgaatggaggaggaaagcaggcccttgaa

A3R14R

ttctcgaggtctcacatagaacaggcaagagtcgctgtacggtttcgagagcttgcttaccacc

A3R14A

ttctcgaggtctcagactcctgaacaggttgtcgccattgcatctaatatcggtggtaagcaagctctcgaa

A3R14C

ttctcgaggtctcagactcctgaacaggttgtcgccattgcatctcacgatggtggtaagcaagctctcgaa

A3R14G

ttctcgaggtctcagactcctgaacaggttgtcgccattgcatctaataatggtggtaagcaagctctcgaa

A3R14T

ttctcgaggtctcagactcctgaacaggttgtcgccattgcatctaatggtggtggtaagcaagctctcgaa

A3R15R

ttctcgaggtctcagcaaagcacaggtaacaacctttgaactgtttcaagagcttgtttgccacc

A3R15A

ttctcgaggtctcatatgccaagcgcatggcttgacgccggaacaggtggtagccatagcaagcaacataggtggcaaacaagctcttgaa

A3R15C

ttctcgaggtctcatatgccaagcgcatggcttgacgccggaacaggtggtagccatagcaagccacgatggtggcaaacaagctcttgaa

A3R15G

ttctcgaggtctcatatgccaagcgcatggcttgacgccggaacaggtggtagccatagcaagcaataacggtggcaaacaagctcttgaa

A3R15T

ttctcgaggtctcatatgccaagcgcatggcttgacgccggaacaggtggtagccatagcaagcaacggtggtggcaaacaagctcttgaa

A3R16R

ttctcgaggtctcagcttgacacaacacgggcaacaggcgctgcactgtctcaagagcaggccgtccccc

A3R16A

ttctcgaggtctcattgccaagcccacggtttgacccctcaacaggtggttgctatagcatcaaatattgggggacggcctgctcttgag

A3R16C

ttctcgaggtctcattgccaagcccacggtttgacccctcaacaggtggttgctatagcatcacatgatgggggacggcctgctcttgag

A3R16G

ttctcgaggtctcattgccaagcccacggtttgacccctcaacaggtggttgctatagcatcaaataatgggggacggcctgctcttgag

A3R16T

ttctcgaggtctcattgccaagcccacggtttgacccctcaacaggtggttgctatagcatcaaatggagggggacggcctgctcttgag

A3R17R

ttctcgaggtctcatcagaccgtgagcttggcacagaacaggcaagagtcgctgaacggtttccagcgcttgtttgccgcc

A3R17A

ttctcgaggtctcaaagcgcatggcttaacaccggaacaggtcgtggcaattgcgtcaaatattggcggcaaacaagcgctggaa

A3R17C

ttctcgaggtctcaaagcgcatggcttaacaccggaacaggtcgtggcaattgcgtcacacgatggcggcaaacaagcgctggaa

A3R17G

ttctcgaggtctcaaagcgcatggcttaacaccggaacaggtcgtggcaattgcgtcaaataatggcggcaaacaagcgctggaa

A3R17T

Ttctcgaggtctcaaagcgcatggcttaacaccggaacaggtcgtggcaattgcgtcaaatggtggcggcaaacaagcgctggaa

**C. Primer sequences for construction of preassembly vectors pL1-TA1-3**

ecvprac1 tttcactctgtggaagacaaaatctgagaccgcagctggcacgacaggtttc

ecvprac11 tttcacttcgtggaagacaataagtgagaccgtcacagcttgtctgtaagcg

ecvprac18 tttcactctgtggaagacaaagaatgagaccgcagctggcacgacaggtttc

ecvprac19 tttcacttcgtggaagacaatcagtgagaccgtcacagcttgtctgtaagcg

ecvprac23 tttcactctgtggaagacaacttatgagaccgcagctggcacgacaggtttc

ecvprac24 tttcacttcgtggaagacaattcttgagaccgtcacagcttgtctgtaagcg
